# Performance of Artificial Intelligence Systems for Automated Segmentation and Quantification of Retinal Fluid and Pathology in Optical Coherence Tomography Scans: A Systematic Review and Meta-Analysis

**DOI:** 10.7759/cureus.108662

**Published:** 2026-05-11

**Authors:** Bakhtawar Awan, Mohamed Elsaigh, Mohamed Hesham Gamal, Sara E Elbahnasawy, Mohammed Badee

**Affiliations:** 1 General Surgery, Northwick Park Hospital, London, GBR; 2 General and Emergency Surgery, Royal Cornwall Hospital Trust, Cornwall, GBR; 3 Pharmacy, Banha University Hospitals, Banha, EGY; 4 Pharmacology and Therapeutics, Faculty of Pharmacy, Tanta University, Tanta, EGY; 5 Diagnostic Radiology, Menofia University Hospital, Menofia, EGY; 6 Ophthalmology, Perfect Vision Eye Hospital, Cairo, EGY

**Keywords:** artificial intelligence, convolutional neural networks (cnn), deep learning, meta-analysis, optical coherence tomography (oct), retinal fluid, systematic review

## Abstract

Optical coherence tomography (OCT) plays a crucial role in diagnosing retinal diseases, such as diabetic retinopathy (DR) and age-related macular degeneration (AMD), as well as in identifying neurodegenerative biomarkers. Despite advancements in U-Net-based convolutional networks for OCT image segmentation, there is a lack of systematic reviews comparing their performance with expert manual segmentations. This review aims to assess the efficacy of these automated networks in segmenting retinal fluid and pathology in OCT images. By searching three different databases, PubMed, Web of Science, and Scopus, over the past five years, we conducted this systematic review and meta-analysis using data from 16 diagnostic-accuracy studies. Study quality was assessed using the Quality Assessment of Diagnostic Accuracy Studies-2 (QUADAS-2) tool. The analysis used mean and standard deviation for the continuous outcomes and employed a random-effects model. Analyses were performed using Review Manager software version 5.4 (The Cochrane Collaboration, London, UK, 2020). Artificial intelligence (AI) and human Dice scores did not differ significantly (standardized mean difference (SMD) = -0.08; 95% CI: -1.16 to 0.99; p = 0.88), nor did intraclass correlation coefficient (ICC) values (SMD = -0.13; 95% CI: -5.70 to 5.45; p = 0.96). However, very high heterogeneity (I² > 90%) limits the reliability of these pooled estimates. AI achieved expert-level Dice scores for subretinal fluid (0.88-0.96) and geographic atrophy (0.94). Intraretinal fluid was more challenging (Dice 0.79-0.89). Volumetric reliability was strong (ICCs > 0.94). Device-dependent variability was substantial; kappa was 0.37 for ZEISS versus 0.73 for Spectralis, indicating a need for device-specific optimization. Volumetric analyses revealed minor systematic overestimation (mean difference: -0.05 mm²). Processing times ranged from 100 milliseconds per B-scan to several seconds per volume, representing substantial time savings versus manual segmentation. Fully automated U-Net pipelines reach expert-level accuracy for subretinal fluid and geographic atrophy but remain limited for intraretinal fluid and show marked device-dependent variability. Clinical translation requires four priorities: standardized multi-device benchmarks, domain adaptation for cross-platform robustness, hybrid AI-human workflows pairing automated pre-segmentation with expert oversight, and prospective clinical trials. These steps are needed to move AI segmentation from a research tool to a clinical decision-support system.

## Introduction and background

In the field of ophthalmology, optical coherence tomography (OCT) plays a crucial role in diagnosing, predicting outcomes, and managing treatments for diabetic retinopathy (DR) and age-related macular degeneration (AMD). This is particularly important, considering that DR is the foremost cause of blindness among working-age individuals in developed nations [1]. Additionally, AMD ranks as the primary cause of central vision impairment in those aged 50 and older across North America and other developed regions [2]. A seven-layer segmentation method for OCT was created employing kernel regression (KR)-based classification to assess diabetic macular edema (DME) and to identify OCT layer boundaries. This technique was integrated with graph theory and dynamic programming approaches. It was validated using 110 B-scans from 10 patients with severe DME, achieving a Dice coefficient of 0.78 [3,4]. OCT is the most frequently used imaging technique in ophthalmology, with approximately 6.74 million examinations conducted in the US Medicare population in 2017. Due to its widespread accessibility, there has been a growing focus on developing a fully automated system for disease identification [5].

Artificial intelligence (AI) has become increasingly integrated into various aspects of daily life, including the field of ophthalmology. AI technologies are used for tasks such as diagnosing conditions, screening patients, assessing disease activity, analyzing treatment responses, and forecasting disease progression or therapy outcomes. A 2016 landmark study demonstrated that deep learning could diagnose DR from color fundus images with high sensitivity and specificity [6]. Subsequent work has extended AI to other retinal conditions, including the detection of polypoidal choroidal vasculopathy using combined OCT angiography (OCTA) and fundus inputs [7]. These early successes established deep learning as a viable approach for ophthalmic image analysis and motivated its application to OCT segmentation [7].

Recent advances in computing capabilities have paved the way for deep learning, an emerging area within machine learning that focuses on convolutional neural networks (CNNs) [8]. CNNs use convolutional layers with learned filter kernels to extract image features [9]. Cicek et al. [10] extended this design to volumetric data with the 3D U-Net, which preserves the characteristic contracting-expansive U-shape and uses skip connections to capture both high-resolution details and broader contextual information, making it well suited to OCT volume segmentation [10]. Research has demonstrated strong diagnostic capabilities of deep learning algorithms combined with OCT in identifying retinal disorders and assessing the urgency of referrals for serious eye conditions that could threaten vision [11-13].

Despite the extensive use of U-Net and its variants for segmenting features in OCT, there is currently no systematic review that evaluates fully automated U-Net-based pipelines across various retinal pathologies, device types, and annotation standards. Previous reviews either provide broad overviews of fully convolutional network (FCN) architecture without conducting pathology-specific meta-analyses or present narrative discussions that lack quantitative comparisons [14,15]. This is a significant gap in evidence-based guidance for clinical implementation and cross-device applicability. This review aims to fill that gap by assessing the diagnostic accuracy of fully automated U-Net-based CNNs for segmenting and quantifying retinal fluid and pathology in OCT images, using expert manual segmentations as a benchmark.

## Review

Methodology

This systematic review was performed following the Preferred Reporting Items for Systematic Reviews and Meta-Analyses (PRISMA) guidelines [16] and Cochrane Handbook for Systematic Reviews of Interventions [17].

Literature Search Strategy

To identify studies on AI-driven automated segmentation and quantification of retinal fluid and pathology in OCT scans, we conducted a comprehensive search of PubMed, Web of Science, and Scopus, covering the last five years. For PubMed, Web of Science we used ("Tomography, Optical Coherence"[MeSH Terms] OR "optical coherence tomography" OR OCT) AND ("Artificial Intelligence"[MeSH Terms] OR "Machine Learning"[MeSH Terms] OR "Deep Learning"[MeSH Terms] OR AI) AND (segmentation OR delineat* OR "image analysis") AND (quantif* OR "volume measurement" OR "volumetric analysis") AND ("Retina"[MeSH Terms] OR retina* OR "retinal fluid" OR "intraretinal fluid" OR "subretinal fluid" OR SRF OR IRF OR lesion* OR biomarker* OR pathology) with filteration to title/abstract for pubmed only. For Scopus, we used TITLE-ABS-KEY (("Tomography, Optical Coherence" OR "optical coherence tomography" OR oct ) AND ( "Artificial Intelligence" OR "Machine Learning" OR "Deep Learning" OR ai) AND (segmentation OR delineat* OR "image analysis" ) AND (quantif* OR "volume measurement" OR "volumetric analysis" ) AND ("Retina" OR retina* OR "retinal fluid" OR "intraretinal fluid" OR "subretinal fluid" OR SRF OR IRF OR lesion* OR biomarker* OR pathology)).

Inclusion and Exclusion Criteria

We applied predefined inclusion and exclusion criteria to ensure relevance and methodological rigor. Eligible studies evaluated adult eyes with retinal fluid or pathology imaged by OCT, used fully automated AI‐based segmentation algorithms, compared AI outputs against expert manual segmentations, and reported metrics such as Dice coefficient, sensitivity, specificity, volumetric error, processing time per scan, or inter‐rater reliability (e.g., intraclass correlation coefficient (ICC)). Studies using complementary modalities (OCTA, fundus autofluorescence (FAF)) were included only when OCT was a primary input to the segmentation algorithm or when OCT-derived biomarkers were the comparator endpoint. We included diagnostic‐accuracy studies, randomized controlled trials, observational, and cohort studies. We excluded non‐human or purely synthetic studies, case reports, reviews, editorials, conference abstracts lacking complete data, and any algorithms requiring manual refinement at inference.

Critical Appraisal

Following PRISMA guidelines and predefined eligibility criteria, two independent reviewers conducted a quality assessment of all included studies. Disagreements between reviewers were systematically resolved through structured discussion and consensus-building until complete agreement was achieved.

Selection of Articles and Data Extraction

After the initial database search, two reviewers independently screened the titles and abstracts of the retrieved articles to identify potentially relevant studies based on predefined inclusion criteria. The full texts of these selected articles were subsequently obtained and assessed for final eligibility by the same two independent reviewers. Any disagreements regarding study inclusion were resolved through discussion and consensus, or by consulting a third reviewer if necessary. Data from the included studies were extracted by the two reviewers using a standardized data extraction form designed specifically for this review. The extracted data encompassed several key components: study characteristics (such as the first author, publication year, study design, and setting), patient demographics (including the number of patients, gender distribution, and mean and standard deviation of age), and imaging details (the number of OCT scans analyzed). We also recorded the architecture of each AI model, which mainly consisted of U-Net variants based on a CNN and other CNN-based approaches.

Additionally, we included information on the size of the training data and the annotation protocols used. The comparator methods involved expert manual segmentations. Performance outcomes assessed included segmentation accuracy metrics (Dice and Jaccard indices), inter-method agreement (ICC), volumetric performance (absolute and percentage error in microliters), processing time per scan, and overall conclusions from the studies. Notably, all reported algorithms functioned as fully automated pipelines during inference, requiring no manual intervention.

Quality Assessment

The methodological quality of the studies included in this review was assessed using the Quality Assessment of Diagnostic Accuracy Studies-2 (QUADAS-2) tool [18], which is a validated framework for evaluating diagnostic accuracy studies. This tool examines four key domains: (1) patient selection, (2) the index test, (3) the reference standard, and (4) flow and timing. Each domain is rated for the risk of bias as high, low, or unclear, while the first three domains are also assessed for applicability concerns, also rated as high, low, or unclear. To guide these evaluations, signaling questions are used, such as, 'Was a consecutive or random sample of patients utilized?' Two reviewers independently rated each domain using the standard QUADAS-2 signaling questions. Inter-reviewer agreement for QUADAS-2 judgments was quantified using Cohen's kappa (κ = [insert]). Discrepancies were resolved through structured discussion, with a third reviewer consulted when consensus could not be reached.

Statistical Analysis

We used RevMan version 5.4 (The Cochrane Collaboration, London, UK, 2020) [19]. Continuous data were analyzed as mean differences (MDs) with 95% confidence intervals (CIs). We applied a fixed-effects model for homogeneous studies and switched to a random-effects model when substantial heterogeneity was detected. Statistical heterogeneity among studies was assessed using I-squared (I²) and chi-squared (Chi²) statistics, with I² values ≥50% considered indicative of high heterogeneity. A leave-one-out sensitivity analysis was conducted for high-heterogeneity plots. Although Dice and ICC are bounded metrics (range 0-1), we used standardized mean difference (SMD) pooling because the included studies reported these metrics on slightly different scales (proportion vs. percentage; varying CI structures). We acknowledge that SMD pooling of bounded metrics may inflate apparent effect sizes when within-study variance is small, and we therefore interpret these pooled estimates as exploratory rather than definitive.

Results

Literature Search and Study Selection

**Figure 1:**
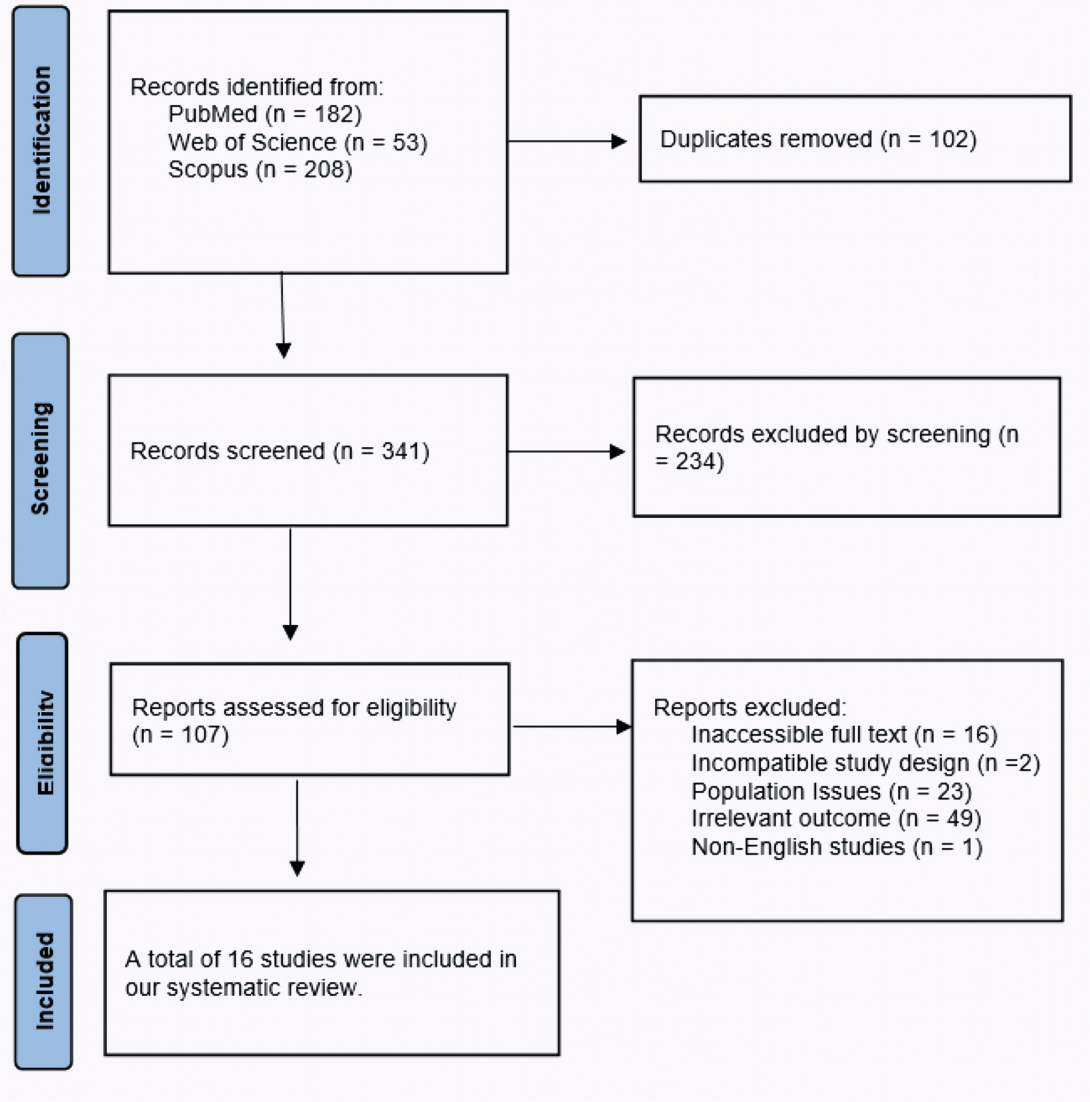
A PRISMA flowchart outlining the study selection process PRISMA: Preferred Reporting Items for Systematic Reviews and Meta-Analyses

Three databases, including PubMed, Web of Science, and Scopus, were searched, yielding 443 papers. After removing 102 duplicates, 16 articles [20-35] were deemed suitable for the systematic review. The full PRISMA flow diagram is shown in Figure 1.

Study Characteristics

Our systematic review and meta-analysis comprised 16 diagnostic accuracy studies involving a total of 6,847 patients. The included studies were conducted across multiple countries, including the Netherlands, Germany, China, the United States, Switzerland, the United Kingdom, and Pakistan, representing diverse healthcare settings from single-center to multinational collaborations. The study designs included both prospective and retrospective approaches, with patient cohorts ranging from 20 to 2,580 participants. The mean age of the included population ranged from 40.9 to 85 years across studies, with the vast majority consisting of elderly patients, reflecting the target demographics for retinal diseases. The studies focused on various retinal pathologies, including neovascular AMD (nAMD), DME, geographic atrophy (GA), and X-linked retinoschisis. OCT scan volumes varied significantly across studies, ranging from 37 B-scans to over 41,000 scans per study. All studies utilized U-Net-based AI architectures and compared their results against manual segmentations performed by expert graders, serving as the reference standard. The training datasets included both institution-specific cohorts and public databases such as the RETOUCH (Retinal OCT Fluid Detection and Segmentation Benchmark Challenge) and Duke datasets. The baseline and summary characteristics of all included studies are presented in Table 1.

**Table 1:** Characteristics of studies evaluating AI-based segmentation of retinal pathologies using optical coherence tomography Abbreviations:AI: Artificial Intelligence; AMD: Age-related Macular Degeneration; AUMC: Amsterdam University Medical Centers; B-scans: Cross-sectional OCT images; CME: Cystoid Macular Edema; CMO: Cystoid Macular Oedema; CNN: Convolutional Neural Network; DME: Diabetic Macular Edema; DR: Diabetic Retinopathy; DRCR: Diabetic Retinopathy Clinical Research; F: Female; FAF: Fundus Autofluorescence; GA: Geographic Atrophy; iAMD: Intermediate Age-related Macular Degeneration; IRF: Intraretinal Fluid; LUMHS: Liaquat University of Medical & Health Sciences; M: Male; nAMD: Neovascular Age-related Macular Degeneration; NR: Not Reported; OCT: Optical Coherence Tomography; OCTA: Optical Coherence Tomography Angiography; OHSU: Oregon Health & Science University; PED: Pigment Epithelial Detachment; RETOUCH: Retinal OCT Fluid Detection and Segmentation Benchmark Challenge; RPE: Retinal Pigment Epithelium; RORA: Reticular Pseudodrusen-Associated Outer Retinal Atrophy; SD: Standard Deviation; SD-OCT: Spectral-Domain Optical Coherence Tomography; SRF: Subretinal Fluid; U-Net: Convolutional Neural Network for Image Segmentation; nnU-Net: No New-U-Net; XLRS: X-Linked Retinoschisis.

First author, year	Study design, setting	Number of patients	OCT scans	Gender M/F (n)	The mean (SD) age (years)	AI model	Training data	Comparator
Almushattat et al., 2025 [20]	Diagnostic-accuracy (prospective) study at the Department of Ophthalmology, AUMC (Amsterdam, The Netherlands)	20 retinitis pigmentosa patients with CMO	37 segmentation performance on single-time-point scans only. Randomly selected: 20; Manually selected: 17	NR	40.9 ± 18.4 years	nnU-Net, 3D segmentation model (v1)	RETOUCH dataset 70 OCT volumes (7 064 annotated B-scans) for training, 42 OCT volumes (4 270 B-scans) for internal validation	Manual by three independent graders
Hensman et al., 2025 [21]	Diagnostic-accuracy (prospective) study at the Department of Ophthalmology, AUMC (Amsterdam, The Netherlands)	20 patients with genetically or clinically confirmed XLRS	37 spectral‐domain OCT B-scans, Single time-point evaluation, Randomly selected: 20, Manually selected: 17	NR	NR	nnU-Net 3D segmentation framework	112 OCT volumes from RETOUCH (70 train, 42 internal test)	Three independent graders manually delineated cystoid fluid collections
Hormel et al., 2025 [22]	Cross‐sectional diagnostic accuracy study at 20 DRCR Retina Network U.S. sites + healthy controls from Oregon Health & Science University ( Portland, Oregon, USA)	375 (101: DR, 274: none)	50 OCTA scans, randomly selected	191/180, 4: NR	57 ± 16	Segmentation U-Net CNN (“MEDnet” style)	Trained on OCTA volumes collected at OHSU (healthy and diabetic eyes) and then tested on the DRCR Retina Network scans.	Manually delineated by a masked grader
Asani et al., 2024 [23]	Diagnostic-accuracy (retrospective) study at Ludwig-Maximilians University Eye Clinic (Munich, Germany) from 2013 to 2020	339 nAMD patients (378 eyes)	458 unique SD-OCT B-scans (one per patient)	144/234	82 ± 8 years	Four-level modified U-Net	458 OCT scans manually annotated (distinct patients); Validation: 84 patients; Test: 36 held-out	Three retinal experts
de Vente et al., 2024 [24]	Diagnostic-accuracy study (prospective) at 20 sites across seven European countries (Denmark, France, Germany, Italy, Portugal, the Netherlands, and the United Kingdom) as part of the MACUSTAR consortium.	168 iAMD patients	Scans: 143; ZEISS OCT volumes, 167 Spectralis OCT volumes	NR	71.2 ± 7.55 years.	U-Net (3D CNN) for 13 biomarkers	Topcon domain: RETOUCH + Rotterdam Study (15,488 B-scans), Spectralis domain: RETOUCH + AUMC (1,622 B-scans)	Manual expert annotations (GRADE Reading Center; discrepancy resolution)
Feng et al., 2023 [25]	Diagnostic accuracy study (prospective) at a single center (Beijing Tsinghua Changgung Hospital, Beijing, China)	69 nAMD with CNV	116 (54 choriocapillaris, 62 outer retina)	40/29	71.3 ± 8.8 years	Modified U-Net with: - ResNeSt blocks (split-attention mechanism) - spatial pyramid pooling	66 OCTA images (44 patients: 22 with both layers, 22 with one layer), Validation/test 50 OCTA images (25 patients with both layers)	Manual segmentation by an expert ophthalmologist
Mishra et al., 2023 [26]	Diagnostic accuracy study (retrospective) at multi-center (ProgStar study consortium, coordinated by the Wilmer Eye Institute, Johns Hopkins University, Baltimore, Maryland, USA)	155 (264 eyes)	264 volumes (100,266 B-scans), Follow-up FAF: 6-month (264 eyes), 12-month (237 eyes)	NR	NR	Ensemble U-Net (modular design)	200 eyes (six-month), 180 eyes (12-month), Training protocol: 60 epochs, batch=8, RMSprop optimizer (LR=0.01→0.001), Loss: weighted logistic + Dice loss	Manual segmentation of atrophy on FAF by certified graders.
Morelle et al., 2023 [27]	Diagnostic accuracy study (retrospective) at the University of Bonn, Bonn, Germany (single center)	Adults with AMD with drusen	21 OCT volumes (five B-scans/volume manually annotated)	NR	NR	Novel CNN with "layer head" (order-constrained regression)	Internal dataset (100 vols) + Duke public dataset (164 vols)	Two independent human readers (expert annotations)
Pawloff et al., 2023 [28]	Retrospective diagnostic accuracy study at	1,185 nAMD patients	41,147 OCT volumes (23,986 from HAWK; 17,161 from HARRIER)	NR	NR	U-Net (encoder-decoder with skip connections).	Training data: Spectralis (Heidelberg) and Cirrus (Zeiss) OCT systems. Generalization: tested on Topcon images	Certified graders at reading centers
Ahmed et al., 2022 [29]	Diagnostic accuracy study (model development/validation) at the Institute of Ophthalmology, LUMHS, Jamshoro, Pakistan	Adults with DME and CME	Training: 800 B-scans (Kaggle DME dataset), Validation: 200 B-scans. - Testing: 200 B-scans (LUMHS), Follow-up: none (cross-sectional).	NR	NR	U-Net with hyperparameter optimization	800 OCT B-scans (Kaggle DME dataset), preprocessed and manually segmented by experts, Loss: binary cross-entropy, Optimizer: Adam, Epochs: 80 (early stopping at the 11^th^ epoch), Preprocessing: minimum filtering, BM3D denoising, Richardson-Lucy deconvolution, contrast adjustment.	
Chu et al., 2022 [30]	Diagnostic accuracy study (prospective) at the University of Miami Miller School of Medicine, Miami, FL, USA (single center)	125 patients with GA secondary to non-exudative AMD	140 eyes (80 GA, 60 normal), Training: 89 eyes (51 GA, 38 normal), Validation: 23 eyes (13 GA, 10 normal), Testing: 28 eyes (16 GA, 12 normal)	14/14	64 ± 22 years (range: 27–93)	U-Net 3-channel false-color OAC en face images	51 GA + 38 normal eyes (89 eyes total), Learning rate: 0.0003, Dropout: 0.3, Batch normalization: momentum=0.1, scale=false, Epochs: 200 (early stopping patience: 50)	Two independent graders outlined GA lesions on en face subRPE OCT images
Kalra et al., 2022 [31]	Diagnostic accuracy study (retrospective) at a single center (Cole Eye Institute, Cleveland Clinic, Cleveland, OH, USA)	341 non-exudative AMD with/without GA	900 volumes (100,266 B-scans; 900 en face images)	NR	NR	U-Net for both B-scan and en face models	Batch size: 40; Epochs: 200 (early stopping after seven epochs without improvement), Loss: binary cross-entropy; Optimizer: RMSprop (LR=1e⁻⁴), Automatic retraining: low-performing patches (F-score <30th percentile) duplicated	Manual segmentation by expert analysts using multi-layer segmentation
Song et al., 2022 [32]	Retrospective diagnostic accuracy study at Qilu Hospital, Shandong University, Jinan City, China	186 patients (186 eyes) nAMD with IRF/SRF/PED	671 B-scans	112/74	75 (11.21)	Fluid segmentation: fully convolutional network with EfficientNet backbone, Photoreceptor Layers: classification CNN	519 scans (148 patients)	Manual segmentation by two ophthalmologists
Derradji et al., 2021 [33]	Diagnostic accuracy study (prospective) at Jules-Gonin Eye Hospital, Lausanne, Switzerland (single center)	57 AMD patients (62 eyes)	Training: 44 eyes (2,557 B-scans), Testing: 18 eyes (1,038 B-scans), Healthy controls: 5 eyes (173 B-scans)	19/38	85 ± 12 years	U-Net (EfficientNet-b3)	44 eyes (2,557 B-scans), RPE/choroid layer masks applied during training, Focal loss (γ=2), five-fold cross-validation	Two independent graders annotated RORA per B-scan
Moraes et al., 2021 [34]	Diagnostic-accuracy (retrospective) study at Moorfields Eye Hospital NHS Foundation Trust, London, United Kingdom (single center).	2,580 nAMD patients	2,966 baseline OCT scans (2,473 first-treated eyes, 493 second-treated eyes).	1131/1835	First-treated eyes: 79.3 (8.6), Second-treated eyes: 81.4 (7.9)	3D U-Net	3D input: processes the entire OCT volume (128 B-scans covering 6x6x2.3 mm), Semantic segmentation: outputs a class label for each voxel in the input volume, Ensemble of five instances: a common technique to improve robustness, where five separate U-Net models are trained, and their predictions are averaged.	Manual binary classification (presence/absence only) by two retinal specialists on a subset of 573 baseline scans for IRF and SRF.
Sappa et al., 2021 [35]	Diagnostic accuracy study (retrospective analysis of prospectively collected scans) at Nanjing Medical University, Nanjing, China	124 patients with AMD with fluid accumulations: IRF, SRF, PED- fluid-filled & non-fluid	B-scans of 124 patients.	NR	NR	RetFluidNet: custom CNN architecture type: encoder-decoder (semantic segmentation)	Source: Cirrus SD-OCT (Carl Zeiss Meditec), Format: 2D B-scans (resized to 256x256 px), Patients: Patient-Dep: All 124 patients' scans pooled & split by image. Patient-Indep: 87 patients (70%) for training. Fluid types: IRF, SRF, PED, combinations, no-fluid scans. Annotated manually.	Manual segmentation by experts ("Ground Truth")

The AI models achieved excellent segmentation accuracy for well-defined pathologies, with subretinal fluid demonstrating the highest performance at Dice scores up to 95.78%, followed by GA at 0.940. Intraretinal fluid demonstrated moderate performance, with Dice scores ranging from 0.79 to 0.89, while challenging pathologies, such as fibrosis, achieved lower F1 scores of 0.85. Inter-method agreement was consistently strong across studies, with ICC exceeding 0.94 for volumetric measurements. Device-specific performance revealed significant variability, with kappa statistics of 0.37 for ZEISS compared to 0.73 for Spectralis OCT systems. Volumetric analyses demonstrated minor systematic biases, including fluid volume overestimation (mean difference: -0.05 mm²) and superior drusen volume correlations compared to manual methods (r = 0.994 vs. 0.984). Central subfield thickness exhibited poor predictive value, with residual errors reaching up to 95.26 µm. Processing times varied from 100 milliseconds per B-scan to several seconds per volume, representing substantial efficiency gains over manual segmentation. The performance metrics revealed AI's clinical readiness for subretinal fluid and GA quantification, while highlighting ongoing challenges for complex pathologies, such as choroidal neovascularization (CNV), and device-dependent optimization requirements. The detailed performance characteristics and clinical implications of all AI algorithms are presented in Table 2.

**Table 2:** Performance metrics of AI-based retinal pathology segmentation in OCT image Abbreviations: AUC: Area Under the Receiver Operating Characteristic Curve, AI: Artificial Intelligence, BM: Bruch's Membrane, CI: Confidence Interval, CME: Cystoid Macular Edema, CNN: Convolutional Neural Network, CNV: Choroidal Neovascularization, CRT: Central Retinal Thickness, CSFT: Central Subfield Thickness, cRORA: Complete Reticular Pseudodrusen-Associated Outer Retinal Atrophy, DA: Domain Adaptation, DC: Dice Coefficient, Dice: Dice Similarity Coefficient, ELM: External Limiting Membrane, ERM: Epiretinal Membrane, EZ: Ellipsoid Zone, F1: F1 Score (harmonic mean of precision and recall), GA: Geographic Atrophy, ICC: Intraclass Correlation Coefficient, ILM: Internal Limiting Membrane, IOU: Intersection over Union, IRF: Intraretinal Fluid, iRORA: Incomplete Reticular Pseudodrusen-Associated Outer Retinal Atrophy, LoA: Limits of Agreement, MAE: Mean Absolute Error, nAMD: Neovascular Age-Related Macular Degeneration, NPA: Non-Perfusion Area, NR: Not Reported, OAC: Offset Attenuation Correction, OCT: Optical Coherence Tomography, OCTA: Optical Coherence Tomography Angiography, PDA: Preretinal Disease Activity, PED: Pigment Epithelial Detachment, RPE: Retinal Pigment Epithelium, RORA: Reticular Pseudodrusen-Associated Outer Retinal Atrophy, SD: Standard Deviation, Sens: Sensitivity, Spec: Specificity, SRF: Subretinal Fluid, VA: Visual Acuity, XLRS: X-Linked Retinoschisis.

First author, year	Segmentation accuracy	Inter-method agreement	Volumetric performance	Conclusion
Almushattat et al., 2025 [20]	Dice score AI randomly sampled B-scans: 0.889 ± 0.010, Centrally selected sampled B-scans: 0.936 ± 0.005, Manually randomly sampled B-scans: 0.878 ± 0.007, Manually selected sampled B-scans: 0.946 ± 0.012	ICC score AI randomly sampled B-scans: 0.945 ± 0.014, Centrally selected sampled B-scans: 0.964 ± 0.011; Manually randomly sampled B-scans: 0.979 ± 0.008, Manually selected sampled B-scans: 0.981 ± 0.011	Slight fluid overestimation, Bland-Altman consistency	The nnU-Net model accurately segments and quantifies cystoid macular edema on SD-OCT in retinitis pigmentosa, matching expert performance and enabling faster, consistent assessments.
Hensman et al., 2025 [21]	Dice score AI randomly sampled B-scans: 0.886 ± 0.010, Manually selected sampled B-scans: 0.936 ± 0.012, Manually randomly sampled B-scans: 0.912 ± 0.014, Manually selected sampled B-scans: 0.946 ± 0.012	ICC score AI randomly sampled B-scans: 0.945 ± 0.014, Manually selected sampled B-scans: 0.964 ± 0.011, Manually randomly sampled B-scans: 0.979 ± 0.008, Manually selected sampled B-scans: 0.981 ± 0.011	Systematic overestimation, Mean diff: -0.05 mm²	The nnU-Net model accurately segments cystoid fluid in XLRS, performing comparably to experts but slightly overestimating cyst area, indicating a need for further XLRS-specific refinement.
Hormel et al., 2025 [22]	IOU: 0.66–0.76, F1 Score: AI: 0.79–0.86, Manual: 0.56-0.64	Innerretinal: AI 0.87 (95% CI 0.15–0.96), Mean (SD) calculated: 0.87 (0.06), Manual: 0.54 (95% CI 0.02–0.78), Mean (SD) calculated: 0.54 (0.06)	NA	AI segmentation outperforms rules-based methods in IOU, recall, F1 score, and correlation with DR severity, though rules-based methods are slightly more precise yet undersegment NPA.
Asani et al., 2024 [23]	F1 Score: SRF: 0.98, Fibrosis: 0.85, RPE: 0.93	NA	12mo Δ: SRF: 0.08→0.01 μm³, mean difference: -0.07 μm³, Fibrosis: 0.02→0.03 μm³, RPE: 0.19→0.18 μm³	The ensemble U-Net achieves expert-level biomarker segmentation, showing major early reductions in IRF, SRF, SRHM, and CRT, with minimal change in PED and RPE. Volumes of IRF, ERM, fibrovascular PED, and fibrosis predict poorer 12-month VA, enabling large-scale biomarker analysis in nAMD.
de Vente et al., 2024 [24]	iRORA/cRORA: Sens: 38.5% (ZEISS), 84.0% (Spectralis), Spec: 93.1% (ZEISS), 93.7% (Spectralis) cRORA (Only Biomarker Detection): Sens: 60.0% (ZEISS), 62.5% (Spectralis), Spec: 96.4% (ZEISS), 97.4% (Spectralis)	κ (3-class): 0.37 (ZEISS), 0.73 (Spectralis), AI cross-device, κ: 0.65, Human cross-device κ: 0.32	AI overestimation (2× manual area in outliers; Bland-Altman trend)	Achieves high specificity (93–97%) for iRORA/cRORA detection with superior cross-device consistency vs. manual grading. DA enables generalization to unseen OCT devices without retraining.
Feng et al., 2023 [25]	AI model AUC of 0.9476 (95% CI: 0.9473–0.9479), accuracy of 0.9891 (95% CI: 0.9889–0.9893), sensitivity of 0.7271 (95% CI: 0.7265–0.7277), specificity of 0.9950 (95% CI: 0.9945–0.9955), an IOU of 0.5867 (95% CI: 0.5864–0.5870), and a Dice coefficient of 0.7299 (95% CI: 0.7295–0.7303), Manual AUC of 1.0, perfect agreement, 100% detection of true CNV pixels, and 100% rejection of non-CNV pixels	NR	NA	The model achieves satisfactory CNV segmentation performance (AUC 0.9476, Dice 0.7299) and outperforms saliency-based/U-Net methods. Limitations include suboptimal sensitivity for small/blurred CNVs and artifacts from retinal fluid. Future work will focus on clinical parameter extraction and larger validation.
Mishra et al., 2023 [26]	AI Dice coefficient of 0.830 for the six-month prediction and 0.823 for the 12-month prediction. Manual perfect agreement	NR		Ensemble U-Net using multiple OCT-derived features (beyond intensity) significantly improves Stargardt atrophy prediction (Dice 0.83 at 6 months). The ELM-IRPE retinal layer is optimal for feature extraction. This approach enables phenotypic differentiation and may guide treatment selection.
Morelle et al., 2023 [27]	Drusen segmentation (Dice score) AI: 0.71 ± 0.16, Manual: 0.67 ± 0.19	AI drusen volumes closer to expert consensus (19/21 volumes). Processing Time: 105 ms/B-scan (Nvidia Titan X GPU).	Volume correlation (r): AI: 0.994 Manual: 0.984	A novel CNN with a “Layer Head” outperforms prior methods in drusen and layer segmentation, improving Dice, correlation, and MAE by preserving anatomical order—ideal for large-scale AMD studies and monitoring.
Pawloff et al., 2023 [28]	Fluid detection AUC Spectralis: IRF 0.93 (HARRIER), SRF 0.91 (HAWK), Cirrus: IRF 0.90 (HARRIER), SRF 0.86 (HAWK)	CSFT correlation (Automated vs. Manual): ILM-to-RPE: ρ=0.942ρ=0.942, ILM-to-BM: ρ=0.819ρ=0.819	Residual error (vs. CSFT) up to 95.26 µm, High variability confirms that CSFT cannot predict AI-quantified fluid volumes.	Validation of 41,147 OCT scans from HAWK/HARRIER shows U-Net segmentation accurately quantifies retinal fluid in nAMD (AUC 0.93 IRF, 0.91 SRF). Unlike CSFT, AI volumetry captures distinct treatment responses, offering superior precision for anti-VEGF management.
Ahmed et al., 2022 [29]	Accuracy: 99.81% (training), 99.78% (validation); Dice coefficient (DC): 87.9% (test set, N = 50 scans); Precision: 88.5% (test); Recall: 87.4% (test); Jaccard index: 97.2% (test).	High correlation between AI and manual fluid quantification	Quantified as pixel counts	The U-Net model achieved high accuracy (99.81%) and Dice coefficient (87.9%) in segmenting CME fluid. Preprocessing (denoising, deconvolution) significantly enhanced performance. The method shows potential for clinical use in monitoring DME progression and reducing manual effort.
Chu et al., 2022 [30]	16 GA eyes in the testing set AI: Dice: 0.940 ± 0.032 Sensitivity: 100% Specificity: 100% Manual: Dice: 1 Sensitivity: 100% Specificity: 100%	NR	NR	The OAC-based model outperforms the OCT subRPE model in GA segmentation, achieving higher DSC, stronger correlation, and lower bias, with enhanced RPE contrast improving deep learning accuracy.
Kalra et al., 2022 [31]	B-scan Accuracy 94%, Sensitivity 90%, Specificity 90%, F-score 0.71, Manual F-score of 1.0, perfect agreement, 100% detection	ICC AI model B-scan: 0.88 (95% CI: 0.82–0.92)	NA	High-performance SD-OCT models achieved 91–96% accuracy for GA and hypertransmission defect segmentation, generalizing across devices. Future work aims to integrate them for pre-GA biomarker analysis.
Song et al., 2022 [32]	IRF: Dice: 0.949 (95% CI: 0.925–0.973), Sensitivity: 0.813 (0.736–0.863), Specificity: 0.998 (0.997–0.998); SRF: - Dice: 0.882 (95% CI: 0.873–0.911), Sensitivity: 0.887 (0.864–0.910), Specificity: 0.998 (0.997–0.998)	NR	NR	The model predicts preretinal disease activity in nAMD, with ELM and EZ preservation as key indicators. These findings advance imaging-based prediction of long-term disease activity and highlight the value of automated photoreceptor layer detection.
Derradji et al., 2021 [33]	AI: Dice score, 0.881 ± 0.074; Kappa, 0.846 ± 0.072; Precision, 0.928 ± 0.054; Recall, 0.850 ± 0.119.Manual: Dice score, 0.863 ± 0.099; Cohen’s kappa (κ), 0.828 ± 0.100; Precision, 0.982 ± 0.014; Recall, 0.782 ± 0.145.	Bland–Altman analysis: AI: Mean bias, −0.58 mm²; limits of agreement (LoA; 95% CI), ±1.98 mm². Manual: Mean bias, −1.39 mm²; limits of agreement (LoA; 95% CI), ±2.23 mm².	NR	AI achieved human-level RORA segmentation with a Dice score of 0.88, compared to an inter-grader Dice score of 0.86. Layer-prior training enhanced recall by 8.5% compared to the model without prior training.
Moraes et al., 2021 [34]	OUT of 573 OCTs IRF agreement rates AI: 354/487 (72.7%), Manual: 487 (85%); SRF agreement rates AI: 473/524 (90.3%), Manual: 524 (91.4%)	NR	First-treated eyes (n=2473) IRF: 0.118 (0.309)/0.007 (0.000–0.090), SRF: 0.455 (0.733)/0.183 (0.022–0.562) Second-treated eyes (n=493) IRF: 0.073 (0.196)/0.003 (0.000–0.049) SRF: 0.258 (0.532)/0.054 (0.006–0.252)	The deep learning algorithm automated OCT feature quantification in nAMD, showing high SRF (90.3%) and moderate IRF (72.7%) agreement with experts. Volumetric data revealed eye, age, ethnicity, and VA differences, underscoring AI’s potential for personalized care and structure–function insights.
Sappa et al., 2021 [35]	AI: IRF: Dice = 78.95%, Overlap = 65.22%, Underestimation = 27.10%, Overestimation = 7.68%; PED: Dice = 90.90%, Overlap = 83.32%, Underestimation = 10.61%, Overestimation = 6.07%; SRF: Dice = 95.78%, Overlap = 91.89%, Underestimation = 6.11%, Overestimation = 2.00%. Manual: IRF: Dice = 80.05%, Overlap = 66.74%, Underestimation = 20.32%, Overestimation = 12.94%; PED: Dice = 92.74%, Overlap = 86.46%, Underestimation = 7.60%, Overestimation = 5.94%; SRF: Dice = 95.53%, Overlap = 91.44%, Underestimation = 5.02%, Overestimation = 3.54%.	NR	NR	RetFluidNet outperformed other models in IRF, SRF, and PED segmentation (Dice: SRF 95.8%, PED 92.7%, IRF 80.1%) without pre/post-processing. SRF was easiest, IRF hardest, with ASPP, skip connections, and hyperparameter tuning key to performance.

Quality Assessment

**Figure 2:**
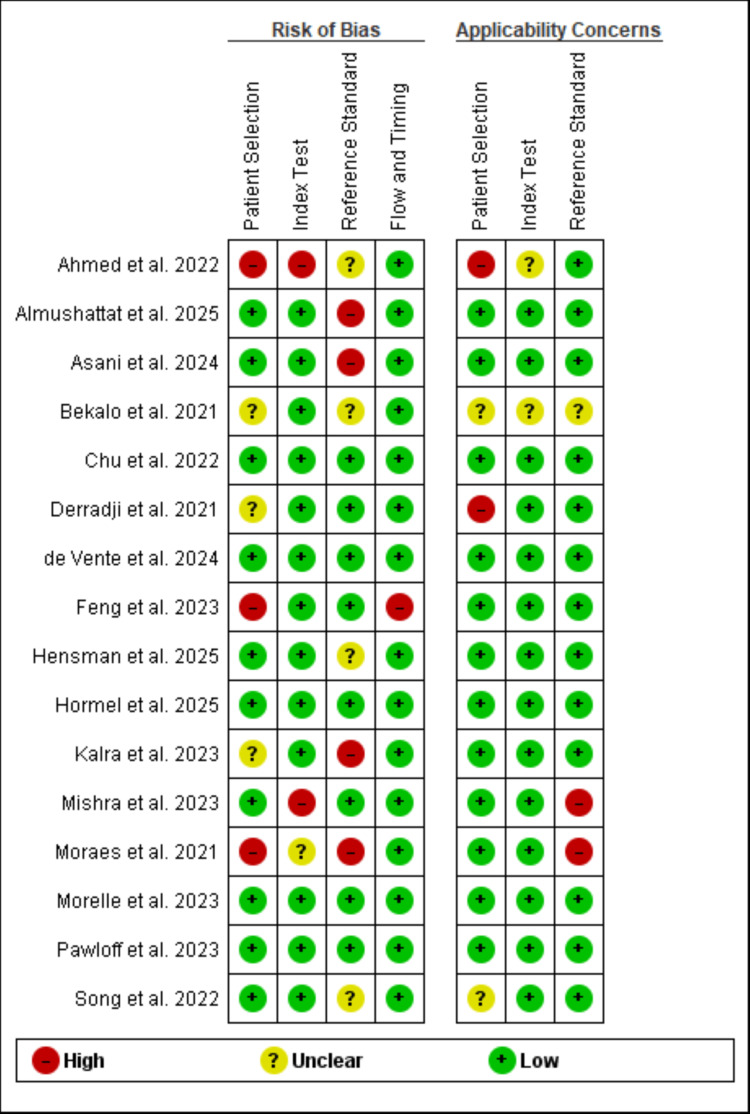
Quality Assessment of Diagnostic Accuracy Studies-2 (QUADAS-2) assessment of the included studies References: [20-35]

The QUADAS-2 Assessment was utilized to evaluate the quality of all diagnostic accuracy studies included in the analysis. Overall, most studies showed a low risk of bias regarding patient selection and the execution of the AI index tests, thanks to well-defined inclusion criteria and blinded evaluations. However, the reference-standard domain occasionally demonstrated a high or unclear risk, particularly in instances where single graders or incomplete annotation protocols were employed. The management of flow and timing was generally effective, although a few studies had overlapping training and test sets or lacked precise timing details. Applicability concerns were minimal for the majority of studies, as the models and cohorts reflected real-world clinical settings. Only a couple of studies raised questions, predominantly due to their focus on rare conditions or the use of heavily preprocessed data. Full data are shown in Figure 2.

Outcomes

**Figure 3:**
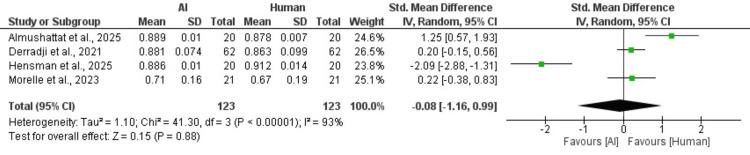
Forest plot of dice scores between Al and human methods References: [20,21,27,33].

Dice scores between AI and human methods: A meta-analysis of four studies comparing Dice scores from AI methods and human methods. The analysis reveals no statistically significant overall difference between the two methods, with a pooled SMD of -0.08 and a 95% CI of -1.16 to 0.99 (p = 0.88), characterized by high heterogeneity (I² = 93%). The heterogeneity remains and can’t be resolved even after conducting a leave-one-out sensitivity analysis (Figure 3).

*ICC*

**Figure 4:**
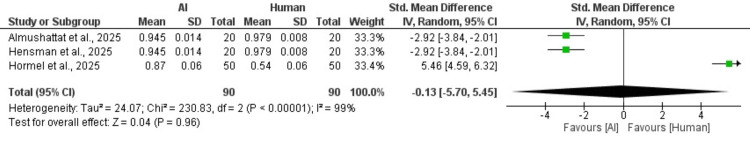
Forest plot between Al and human methods for intraclass correlation coefficient (ICC) References: [20-22]

A meta-analysis of three studies that compared ICC values from AI methods and human methods. The analysis revealed no significant overall difference between the two approaches (pooled SMD = -0.13, 95% CI: -5.70 to 5.45, p = 0.96). However, there was a high level of heterogeneity among the studies (I² = 99%) (Figure 4).

Discussion

Summary of Findings on Fully Automated AI Models

Our systematic review of 16 diagnostic accuracy studies highlights the diverse range of fully automated U-Net-based pipelines employed for OCT segmentation across various retinal pathologies. The study employed multiple models, including the nnU-Net framework, which has proven effective for accurately segmenting 3D cysts in conditions such as retinitis pigmentosa and X-linked retinoschisis [20,21]. Additionally, custom architectures, such as RetFluidNet, were utilized for distinguishing between multiple fluid types [35]. The research also explored hybrid Attention U-Nets for uterine OCT, highlighting the expanding scope of the field. The training datasets varied, ranging from institution-specific cohorts, such as RETOUCH and Moorfields, to multisite collections from consortia like MACUSTAR and the DRCR Retina Network. The number of B-scans included in the studies ranged from 37 to over 41,000 [20-35]. Despite this variation, all models operated fully automatically, requiring no manual adjustments during the inference process. They utilized skip connections and encoder-decoder structures typical of U-Net variants.

Deep Learning for OCT Segmentation

Numerous techniques utilizing deep learning for OCT segmentation focus on identifying the positions of retinal layers and abnormalities, such as edema. These methods typically address the problem through per-pixel classification, resulting in semantic segmentation. A significant number of these strategies employ FCNs, which assign the most likely retinal layer label to each pixel in the image. FCNs are a specific type of ConvNet designed to accept tensor-like input data and produce tensor-like outputs [36,37]. These networks can transform 2D or 3D OCT images into corresponding labeled 2D or 3D images, where each pixel or voxel is associated with a specific class label. One of the most widely used FCNs is the U-Net [38]. Therefore, our review aimed to evaluate the performance of fully automated U-Net-based CNNs for segmenting and quantifying retinal fluid and pathology in OCT images, compared against expert manual segmentations.

High-Accuracy Subretinal Fluid Segmentation

Subretinal fluid segmentation emerged as the most reliably captured pathology. Across studies, Dice coefficients for subretinal fluid ranged from 0.89 ± 0.01 in nnU-Net models to 0.9578 in RetFluidNet, with ICC exceeding 0.94 for cyst quantification [20,35] and area under the curve (AUC) values above 0.90 in large‐scale trial data [28]. These results support previous findings that high-contrast, well-defined fluid pockets are optimal targets for U-Net segmentation, as noted in Pekala et al.’s [39] review of OCT deep-learning methods. The application of machine learning approaches for the analysis of retinal layers and lesions observed in OCT has shown great potential in enhancing diagnostic processes. Recent advancements, particularly through deep learning techniques that largely utilize FCNs, have led to notable improvements in the automated segmentation of OCT images [39]. Performance differences in segmentation accuracy across various pathologies, particularly the stronger performance in cases of subretinal fluid compared to GA, result from both technical and biological factors. Subretinal fluid presents a distinct hyporeflective pocket against the hyperreflective retinal pigment epithelium (RPE), creating sharp interfaces that U-Net models can easily learn. In contrast, GA involves a diffuse degeneration of the RPE and outer retinal layers. This condition is characterized by subtle gradients of reflectance without clear boundaries, making automated delineation more difficult. From a biological standpoint, the distinct fluid-tissue interfaces in subretinal fluid offer strong, high-contrast features that CNNs can effectively utilize. On the other hand, the gradual atrophic changes in GA require the detection of nuanced texture variations and intensity gradients, presenting a greater challenge for segmentation algorithms [40,41].

Variability in Intraretinal Fluid Segmentation

Intraretinal fluid segmentation proved more variable. Dice scores dropped to 0.79 in U-Net models without attention modules. In contrast, ensemble approaches, such as those by Asani et al. (2024) [23] and Mishra et al. (2023) [26], improved intraretinal fluid detection to above 0.83, suggesting that multi-scale feature aggregation and split-attention blocks can partially mitigate the challenges of low-contrast lesions. However, agreement rates for intraretinal fluid presence versus manual grading ranged from 72.7% to 81.3% across cohorts [32,34], underscoring the persistent difficulty of segmenting diffuse, small-scale fluid components.

*GA and Reticular Pseudodrusen-Associated Outer Retinal* Atrophy (RORA)*: Device-Dependent Segmentation Performance*

GA and atrophy subtypes (incomplete RORA (iRORA)/complete RORA (cRORA)) were segmented with high specificity (up to 97%). Dice scores approaching 0.94 [30], yet sensitivity varied widely by device, 38.5% on ZEISS vs. 84.0% on Spectralis in the MACUSTAR consortium [24]. The κ gap between Spectralis (0.73) and ZEISS (0.37) scans is due to device-specific factors. Spectralis benefits from frame averaging and enhanced-depth imaging, resulting in better image quality and delineation of fluid-tissue interfaces. Conversely, ZEISS uses a sparser 200×200 B-scan raster, leading to lower resolution and more noise. These issues affect the U-Net segmentation model, which isn’t adapted for ZEISS, and introduce biases that further lower the κ value for ZEISS compared to Spectralis [24]. Attention-augmented and residual U-Net variants measured in studies by Kalra et al. 2022 and Derradji et al. 2021 [31,33] achieved Dice >0.88 and kappa >0.84 for RORA, indicating that transformer-inspired modules and layer‐prior training boost performance on low‐contrast atrophy regions. These device‐specific discrepancies echo concerns in Kugelman et al.’s [42] comparative study, which found minimal benefit from complex U-Net variants for layer segmentation but highlighted the necessity of harmonized datasets for fair evaluation (2022). This research indicates that the standard U-Net architecture is adequate for segmenting retinal layers in OCT images. It suggests that advanced techniques and variations may not be necessary for this specific task, particularly given the added complexity and slower processing speeds that result in only slight performance improvements. Since the U-Net and its adaptations are widely used for image segmentation, the consistent results observed across multiple datasets are likely applicable to many other OCT datasets and investigations. This insight is highly valuable, as it can save time and reduce costs in experimental setups and model development, while also leading to faster inference times in practical applications by opting for simpler models [40]. Implementing automated OCT segmentation pipelines incurs various costs, including software licensing, hardware, and personnel training. Ongoing maintenance and subscription fees also contribute to expenses. However, the potential savings from reduced manual segmentation and faster image analysis can lead to a positive return on investment.

Pathology-Specific Extensions

Pathology-specific explorations extended to CNV [25], where an AUC of 0.9476 and a Dice score of 0.7299 demonstrated moderate success in detecting irregular CNV lesions amidst fluid artifacts. Stargardt atrophy prediction using ensemble U-Nets (Mishra et al. 2023) [26] secured Dice ≈ 0.83 at both six and 12 months, while drusen segmentation via novel “Layer Head” CNNs yielded Dice = 0.71 and volume correlations r = 0.994 [27]. Automated CME detection in DME achieved 99.8% accuracy but plateaued at Dice ≈ 0.88 without further refinement [29]. For nonperfusion areas in OCTA, U-Net-based AI outperformed rule-based algorithms in F1 (0.79-0.86 vs. 0.56-0.64) and recall (0.87 vs. 0.54), confirming deep learning’s superiority for vascular metrics [22].

Aligning Findings with Previous U-Net Reviews and Meta-Analysis Outcomes

A meta-analysis of three studies compared ICC values from AI methods and human methods. The analysis found no significant overall difference between the two approaches, with a pooled SMD of -0.13 (95% CI: -5.70 to 5.45, p = 0.96). However, the very high heterogeneity (I² = 99% for ICC; I² = 93% for Dice) substantially limits the reliability of these pooled estimates. These results should be interpreted as indicating that current evidence is insufficient to demonstrate a consistent difference between AI and expert human segmentation, rather than as definitive proof of equivalence. There were discrepancies in findings between Hormel et al. (2025) [22], Almushattat et al. (2025) [20], and Hensman et al. (2025) [21] that stem from fundamental differences in their segmentation targets and methodologies. Hormel et al. [22] demonstrated that AI performed exceptionally well in detecting diffuse non-perfusion areas in DR, employing advanced deep learning techniques. The AI achieved higher recall but lower precision compared to rule-based methods. In contrast, Almushattat [20] and Hensman [21] emphasized the strengths of human graders in delineating well-defined cystoid fluid cavities in inherited retinal diseases. Here, the AI's tendency to overestimate fluid areas resulted in lower agreement with human experts. The variation in performance metrics, ranging from recall and precision to image quality dependence, highlights the distinct challenges in these segmentation tasks. Hormel's [22] AI focuses on detection, while human graders excel in precise boundary definition.

In addition, a meta-analysis of four studies comparing Dice scores between AI methods and human grading approaches found no statistically significant difference overall, with a pooled SMD of -0.08 (95% CI: -1.16 to 0.99, p = 0.88). Notably, the findings from Hensman et al. [21] revealed a significant gap between the AI's Dice score of 0.886 and the human graders' score of 0.912 when analyzing B-scans, particularly indicating challenges with central slices. Four key factors contributed to this observed discrepancy: a mismatch between the training and testing domains, as the AI was primarily trained on data from AMD and retinal vein occlusion (RVO); sampling issues that involved many low-contrast slices, leading to boundary errors; inherent variability in microcysts in X-linked retinoschisis, which complicates segmentation; and a lack of domain adaptation for Spectralis test data. However, when the analysis focused specifically on central B-scans, where cysts were most pronounced, the AI’s performance showed significant improvement (0.936 vs. 0.946), indicating that the challenges faced were more a result of domain shift and sampling discrepancies rather than fundamental limitations of the AI system.

Our findings corroborate and extend prior narrative reviews of OCT segmentation [39, 40, 43], which have emphasized the U-Net’s dominance but noted the marginal gains of complex variants. The comprehensive U-Net review demonstrated that despite the ongoing challenges in image analysis, the U-Net architecture has considerable innovative potential and value in analyzing and processing medical datasets. Future advancements in network designs based on the U-Net framework are expected to greatly enhance performance [41]. Unlike those broad overviews, our meta-analysis of pathology-driven studies highlights that ensemble strategies, attention modules, and domain adaptation can yield clinically meaningful improvements in challenging tasks such as intraretinal fluid, CNV, and GA segmentation. However, high heterogeneity (I² > 90%) persists in meta-analytic comparisons of Dice and ICC, reflecting the need for transparent reporting of imaging protocols, annotation methods, and hyperparameter tuning across studies.

Future Directions for AI-Based OCT Analysis

In summary, while U-Net-based pipelines rival expert graders for well-defined lesions such as SRF and GA, their performance on subtle, low-contrast pathologies remains variable. Future work should prioritize cross‐device validation, federated or transfer learning to enhance generalizability, and hybrid AI-human workflows where automated pre-segmentation is followed by expert refinement. A dedicated systematic review of such collaborative approaches will be essential to chart the next phase of AI integration in retinal OCT interpretation.

Positioning Within the Broader AI-in-Imaging Landscape

Our findings should be considered alongside complementary evidence on AI integration in medical imaging. A parallel systematic review on hybrid AI-human workflows for OCT retinal pathology quantification demonstrated that combining AI segmentation with clinician oversight achieves expert-level reliability for 11 of 13 retinal biomarkers, with processing time reductions exceeding 50% compared with manual grading [44]. This evidence reinforces our conclusion that hybrid AI-human pipelines, rather than fully autonomous systems, represent the most realistic near-term pathway for clinical translation - particularly for low-contrast pathologies such as intraretinal fluid and sub-RPE lesions, where fully automated U-Net performance remains variable. More broadly, evidence from AI applications in oncologic imaging indicates that AI-assisted systems consistently outperform conventional diagnostic methods across modalities, but most studies lack external validation across diverse populations, limiting generalizability [45]. The same caveat applies to OCT segmentation: the predominance of single-center retrospective studies, heterogeneous annotation standards, and limited cross-device validation in our included literature underscores that prospective multicenter validation remains the critical next step before clinical adoption.

Strengths and Clinical Implications

Our review's primary strength lies in being the first comprehensive analysis focused on fully automated U-Net-based and related CNN models applied to real-world OCT datasets. We evaluated 16 high-quality studies on diagnostic accuracy published between 2021 and 2025. By systematically extracting and synthesizing performance metrics, such as Dice and Jaccard coefficients, sensitivity and specificity, ICCs, volumetric errors, and processing times, we provide a detailed, pathology-specific comparison that surpasses previous narrative overviews.

The implications of our findings are significant. Clinically, our demonstration that U-Net variants reliably segment subretinal fluid and GA supports their integration into automated OCT workflows, streamlining biomarker quantification in clinical trials and routine patient care. By identifying performance gaps, particularly for intraretinal fluid, CNV, and across different OCT devices, we provide insights that can guide priorities for domain-adaptation research and standardized multi-device benchmarking efforts. Moreover, we highlight the potential of ensemble models and attention modules, creating a roadmap for AI developers to refine architectures for handling challenging, low-contrast lesions. Ultimately, our recommendation for hybrid AI-human segmentation approaches sets the stage for future studies to evaluate collaborative workflows that combine algorithmic speed with expert oversight, thereby accelerating the translation of these technologies into practice and enhancing patient outcomes.

Application of AI in Clinical Settings

The implementation of U-Net-based OCT segmentation models within clinical settings necessitates meticulous planning and adherence to regulatory standards, including obtaining FDA approval. Ensuring compliance with established safety and efficacy criteria is crucial to effectively mitigate clinician workload. The performance of these models in real-world clinical environments is of paramount importance, particularly in addressing challenges such as motion artifacts and comorbid pathologies that may affect segmentation accuracy. Moreover, variability in imaging quality can influence clinicians' trust in the technology. To enhance acceptance among medical professionals, the integration of interpretability tools, such as overlay visualizations and confidence maps, is recommended, as these tools provide essential transparency regarding the segmentation outcomes. Preliminary studies indicate a significant reduction in image reading times, ranging from 50% to 70%. However, to substantiate these findings, prospective clinical trials are essential. Such trials should focus on evaluating the impact of automated segmentation on diagnostic accuracy, as well as its implications for treatment decisions and patient outcomes in routine clinical practice.

Future Recommendations

To enhance the advancement and clinical adoption of automated OCT, several priority actions are proposed. Firstly, the development and adoption of standardized protocols for image preprocessing, annotation guidelines, and performance evaluation are essential across multi-center collaborations. Furthermore, future models should be trained and validated on datasets from multiple vendors to ensure device-agnostic generalizability. It is also critical to conduct prospective, randomized clinical trials to assess the benefits of automated segmentation on workflow efficiency, diagnostic decision-making, and patient outcomes. Additionally, comprehensive cost-benefit analyses in real-world settings will provide valuable insights for budget planning and investment strategies. The integration of explainable AI techniques, such as saliency mapping and uncertainty estimation, can further bolster clinician trust. Lastly, the establishment of open data repositories containing anonymized OCT volumes and ground-truth reference annotations will facilitate benchmarking and foster collaborative innovation in the field.

Limitations

Despite these contributions, our review acknowledges several limitations. First, the significant heterogeneity (I² > 90%) arises from variations in OCT platforms, scan resolutions, preprocessing methods, annotation protocols, and hyperparameter tuning, making direct comparisons of pooled metrics challenging. Second, nearly half of the included studies are retrospective or based on specialized datasets, which raises concerns about the performance of AI in real-world clinical settings. Additionally, reporting gaps, such as inadequate blinding, reliance on single-grader reference standards, and overlapping training/test cohorts, may introduce bias, inflating AI performance estimates. Furthermore, our meta-analyses were limited by the small number of studies providing comparable Dice or ICC statistics, restricting statistical power and subgroup analyses by device type or pathology. Lastly, the potential for publication bias toward positive AI findings should not be overlooked, given the absence of registry data for diagnostic-accuracy studies in ophthalmic imaging. Addressing these limitations will require prospective, multicenter validations with standardized, publicly available benchmarks and transparent reporting guidelines.

## Conclusions

Fully automated U-Net-based models have demonstrated expert-level accuracy in key OCT segmentation tasks, especially in quantifying subretinal fluid and geographic atrophy. However, variability across fluid subtypes, imaging devices, and annotation standards hinders direct clinical translation. To advance the field, several recommendations can be made: first, the development of publicly available, multi-device benchmark datasets with standardized annotation protocols and multi-grader adjudication is essential. Additionally, implementing domain-adaptation and ensemble learning techniques will help enhance cross-platform robustness. Rigorous study designs that incorporate blinded evaluations, non-overlapping training and testing cohorts, and transparent reporting of flow and timing are crucial. Furthermore, prospective trials that evaluate hybrid AI-human workflows, where AI conducts initial segmentation, and experts provide targeted refinements, should be prioritized. Lastly, a dedicated systematic review of these hybrid approaches is necessary to quantify improvements in efficiency, accuracy, and clinical acceptance. By addressing these areas, future research can solidify AI’s role in OCT interpretation and pave the way for integrated, hybrid intelligence solutions that leverage the strengths of both algorithms and human expertise.
